# Metabolic reprogramming in ulcerative colitis: integrating dietary drivers, pathogenesis, and therapeutic advances

**DOI:** 10.3389/fimmu.2026.1894784

**Published:** 2026-09-15

**Authors:** Yunze Liu, Yisha Wan, Xinyu Lu, Aili Xu, Xia Ding, Yuan Li

**Affiliations:** 1 National Institute of TCM Constitution and Preventive Medicine, Beijing University of Chinese Medicine, Beijing, China; 2 The First Clinical Medical College, Xuzhou Medical University, Xuzhou, China; 3 Dongzhimen Hospital, Beijing University of Chinese Medicine, Beijing, China; 4 Wangjing Hospital, China Academy of Chinese Medical Sciences, Beijing, China; 5 School of Traditional Chinese Medicine, Beijing University of Chinese Medicine, Beijing, China

**Keywords:** diet, epidemiology, mechanisms, metabolic reprogramming, ulcerative colitis

## Abstract

Ulcerative colitis (UC) is a complex disorder characterized by gene–environment interactions that compromise the integrity of the mucosa and submucosa. The current evidence indicates a significant correlation between the increasing incidence of UC and the consumption of a Western diet. Dietary patterns alter the nutritional composition, thus inducing metabolic shifts in the intestinal microenvironment that disrupt gut homeostasis and contribute to the development of UC. Mechanistically, metabolic abnormalities in epithelial cells, macrophages, neutrophils, and other cell types compromise the integrity of the intestinal epithelial barrier and modulate immune–inflammatory responses, promoting the onset and progression of UC. Furthermore, the dysregulation of microbial metabolites disrupts gut microbiota homeostasis, contributing to disease progression. Here, we summarize the association between the dietary pattern and UC risk; we further elaborate on the metabolic alterations in colonic epithelial cells, immune cells, and the gut microbiota during UC; and we discuss how these changes influence disease progression. Finally, we discuss potential therapeutic strategies targeting metabolic reprogramming for UC management. By integrating insights from macroepidemiology and micromolecular mechanisms, we delineate the association between metabolic reprogramming and UC and provide a valuable reference for future research into key mechanisms and novel treatment approaches.

## 1 Introduction

Ulcerative colitis (UC) is an inflammatory bowel disease of unknown etiology, and its incidence is continuously increasing worldwide. Although UC is traditionally more prevalent in Western regions, the incidence of this disease has rapidly increased in recent decades in the newly industrialized countries of East Asia (1–3). The 2025 ACG clinical guidelines indicate that the incidence of UC is approximately 6.3 new cases per 100,000 individuals per year, with an estimated prevalence of 305 cases per 100,000 people; based on the 2020 census data, the total number of affected individuals has reached 1.253 million (4).

In terms of pathogenesis, UC is mediated by a combination of genetic susceptibility and environmental factors, and it primarily affects the colorectal mucosa and submucosal tissues and leads to structural and functional impairments. UC is traditionally viewed as an autoimmune-associated mucosal lesion; however, it is increasingly recognized as being closely linked to chronic metabolic disorders. Further studies revealed that both UC and metabolic syndrome share underlying pathological mechanisms involving chronic inflammation and immune dysregulation (5–7), which are common features associated with intestinal metabolic disturbances induced by a Western-style diet (high in fat and sugar and low in fiber) (8, 9). These metabolic disturbances not only promote the development of metabolic syndrome but also drive metabolic reprogramming in immune cells, which is defined as an adaptive shift in metabolic patterns in response to metabolic stress. This reprogramming process directly participates in regulating intestinal immune responses; for example, adiponectin alleviates inflammation by suppressing Th17 cell differentiation and promoting regulatory T-cell generation (10, 11), whereas leptin increases T-cell proliferation and activation and amplifies macrophage-mediated inflammatory responses, in turn exacerbating the inflammatory cascade (12, 13). Thus, metabolic reprogramming serves as a critical link between metabolic abnormalities and immune dysregulation, collectively contributing to the pathogenesis of UC.

Additional evidence indicates that metabolic reprogramming plays a key role in the development and progression of UC through the following mechanisms: influencing damage to and the repair of the colonic epithelium via various metabolic factors and related signaling pathways; regulating the functional states of immune cells such as macrophages, neutrophils, and T cells; and consequently modulating intestinal immune responses. Moreover, microbe-derived metabolites such as short-chain fatty acids (SCFAs) and lactate also play important roles in reshaping the structure and function of the gut microbiota (14, 15). Therefore, metabolic reprogramming not only closely links environmental factors such as the diet to the pathogenesis of UC but also serves as a central integrative mechanism across multiple levels, including colonic epithelial repair, immune response regulation, and host–microbial metabolic interactions (**Figure 1**).

**Figure 1:**
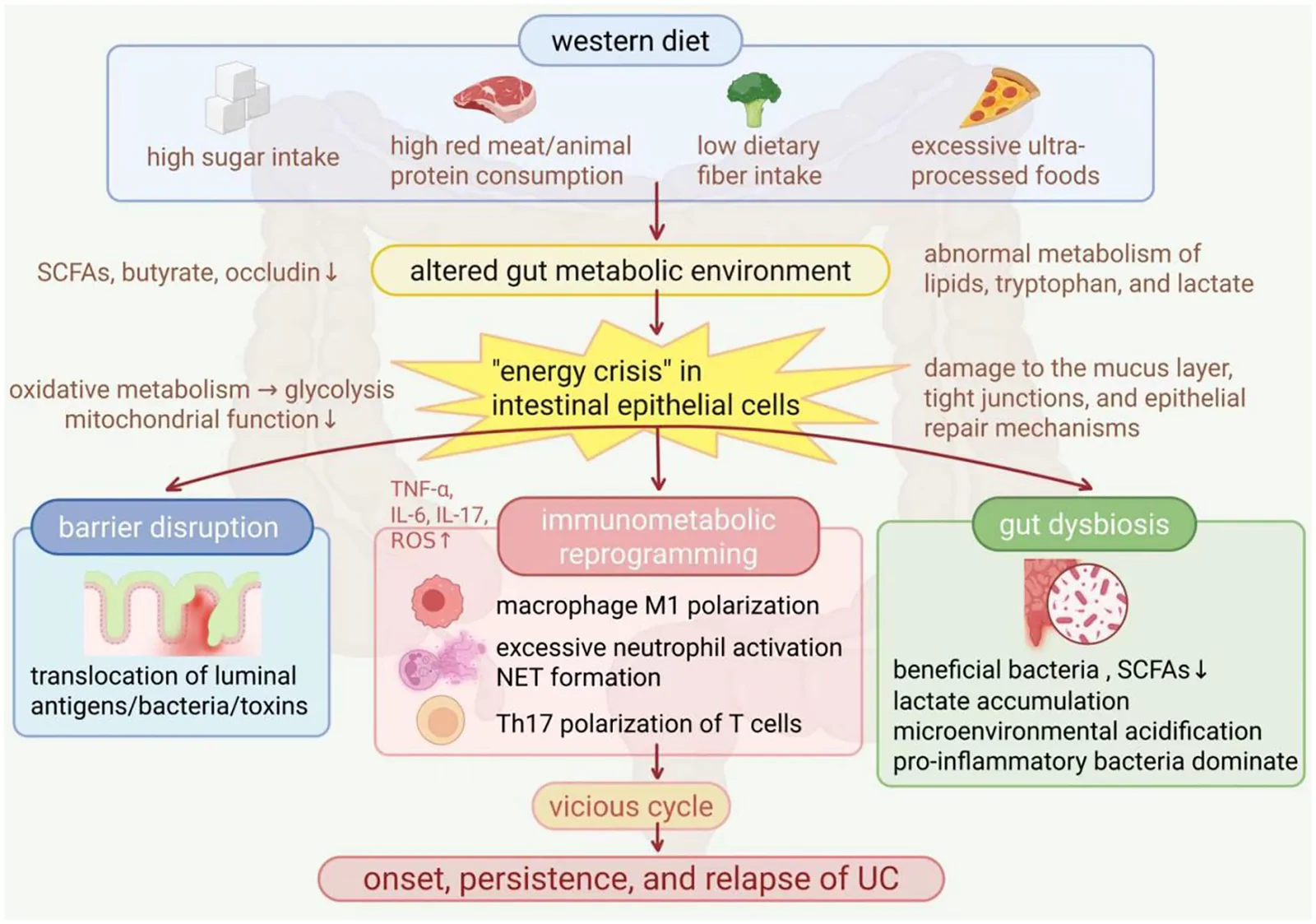
A Western diet promotes UC through a vicious cycle. This diagram depicts how a Western diet, which is high in sugar, red meat, and ultra-processed foods and low in fiber, disrupts intestinal metabolism and reduces butyrate levels. The resulting energy crisis in epithelial cells impairs barrier function, leading to bacterial and toxin translocation. This process activates immune reprogramming and gut dysbiosis, which is characterized by a loss of beneficial bacteria, lactate accumulation, and a predominance of proinflammatory microbes. These events reinforce one another, creating a vicious cycle that drives the onset, persistence, and recurrence of UC. Some graphical elements in this figure were created using BioGDP.com (https://BioGDP.com) (16).

Given this mechanistic background, this review first introduces the evidence linking dietary–metabolic patterns to UC from both macroepidemiological and microepidemiological perspectives; then, it discusses the role of metabolic reprogramming in UC and provides an in-depth analysis of metabolic alterations in colonic epithelial cells, immune cells, and the gut microbiota in UC from a microepidemiological perspective, offering new insights into the multidimensional relationship between metabolic reprogramming and the complex pathogenesis of UC. Finally, this study explores UC treatment strategies based on metabolic reprogramming, providing an important theoretical foundation and reference for the development of more effective therapeutic approaches for UC.

## 2 The dietary–metabolic axis: from epidemiological clues to pathogenic mechanisms

Nutrients such as sugars, lipids, and amino acids are ingested and transported into the body via dietary intake, where they undergo catabolic and anabolic metabolic processes involving complex mechanical and chemical transformations. For instance, monosaccharides—simple carbohydrates—are absorbed by small intestinal epithelial cells via glucose transporter 4 (GLUT4), enter the liver through the portal vein for metabolism, and are subsequently stored as glycogen or lipids for energy. In contrast, poorly absorbable complex carbohydrates, such as dietary fiber, are fermented by the gut microbiota in the colon into SCFAs. Dietary lipids are absorbed by enterocytes through either diffusion in a concentration gradient or active transport and are assembled into chylomicrons. These chylomicrons, which serve as energy carriers, enter the liver via the lymphatic and circulatory systems, where they are metabolized, stored, or distributed. The types and abundance of dietary components influence the host’s nutritional status and metabolic activity (17).

Alterations in the metabolic landscape of the gut microenvironment may disrupt intestinal homeostasis and have been implicated in the initiation and progression of UC (**Figure 2**). Current epidemiological and molecular studies provide substantial evidence that dietary and metabolic factors are associated with the development and progression of UC (**Table 1**).

**Figure 2:**
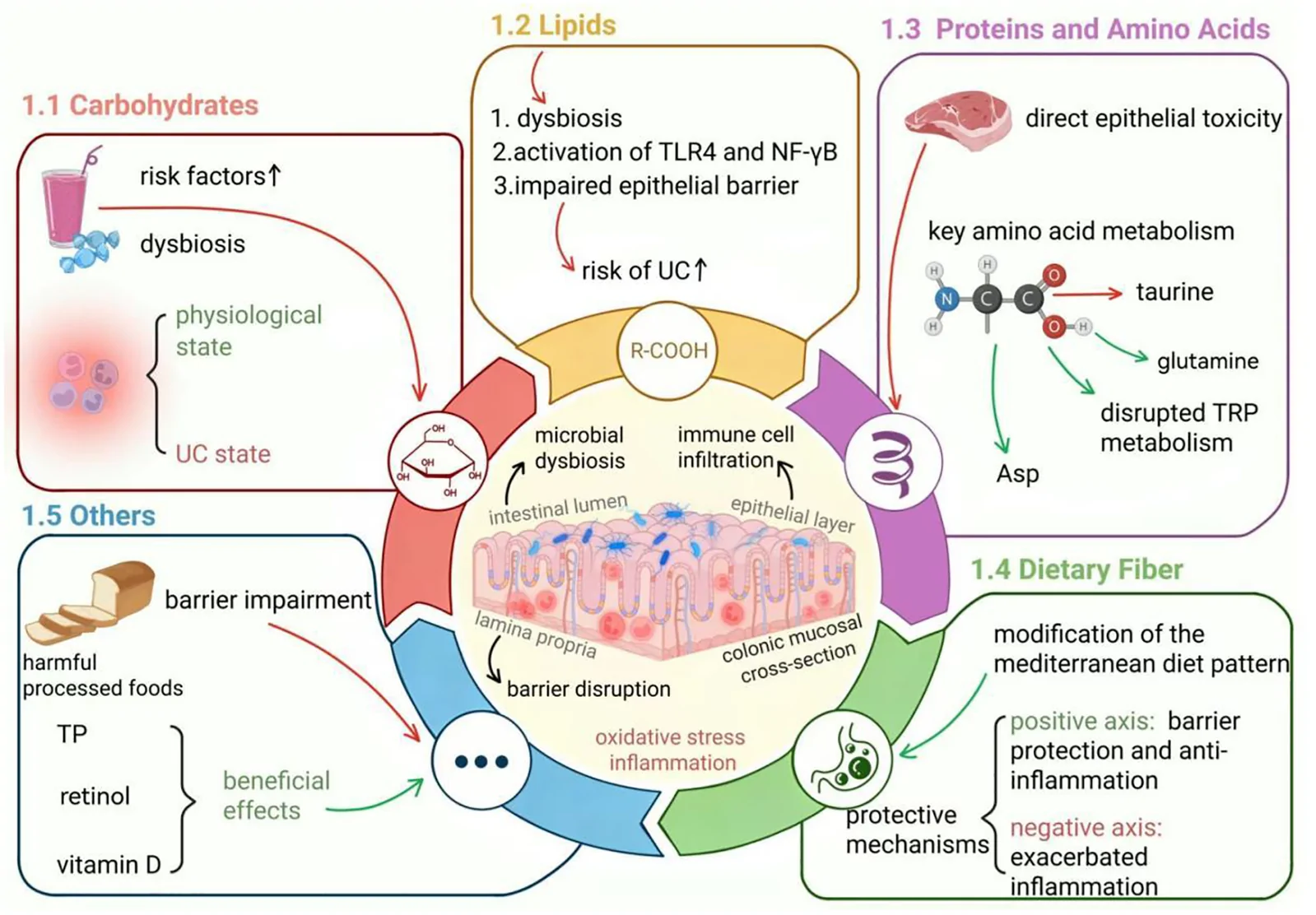
Mechanism by which dietary–metabolic disruption drives UC. 1.1 High intake of sugar-rich foods/drinks is associated with an increased UC risk. 1.2 Excess consumption of trans fats, AA, and an HFD induce dysbiosis, activate TLR4/NF-κB signaling, downregulate tight junction proteins, and impair barrier integrity. 1.3 Undigested proteins are fermented to produce toxic H_2_S; the dysregulated metabolism of tryptophan, glutamine, and taurine impairs immune homeostasis and promotes inflammation, potentially contributing to UC. 1.4 Fiber-rich diets yield SCFAs that upregulate tight junction proteins and inhibit apoptosis, protecting the barrier; low-fiber conditions cause mucus erosion and injury. 1.5 Sweeteners, emulsifiers, and AGEs impair barrier function; tea polyphenols (antioxidants/anti-inflammatory agents), retinol (immune regulators), and vitamin D (inhibits ferroptosis via ACSL4 downregulation) exert protective effects. Some graphical elements in this figure were created using BioGDP.com (https://BioGDP.com) (16).

**Table 1:** Epidemiological evidence of the associations between dietary and metabolic factors and UC.

Dietary–metabolic factors	Substance category	Reported association or effect in UC-related studies (↑=increased risk, ↓=decreased risk)	Human studies	Animal studies	Mechanistic studies	References
Sugar-sweetened soft drinks	Carbohydrates	↑	✓			(10)
Sucrose	Carbohydrates	↑	✓			(11)
Arachidonic acid	Lipids	↑	✓			(13)
Trans unsaturated fatty acids	Lipids	↑	✓			(14)
n-3 PUFAs	Lipids	↓	✓	✓	✓	(18, 19)
HFD	Lipids	↑	✓	✓	✓	(20, 21)
Meat (especially red meat)	Proteins	↑	✓			(22)
FTO	Proteins	↑		✓		(23)
ID2	Proteins	↓	✓	✓		(24)
Legumes	Proteins	↓	✓			(22)
TRP	Amino acids	↑		✓	✓	(25)
KMO and KYNU	Amino acids	↑		✓		(26)
Taurine	Amino acids	↓	✓			(27)
Aspartic (potential role)	Amino acids	↑		✓		(28)
Aspartate supplementation (experimental colitis model)	Amino acids	↓		✓		(28)
Fruits	Dietary fiber	↓	✓			(26)
Vegetables	Dietary fiber	↓	✓			(29)
Sweet additives	Dietary fiber	↑	✓			(28)
Lignin	Dietary fiber	↓		✓		(30)
QBSDF	Dietary fiber	↓		✓		(31)
HPIDF	Dietary fiber	↓		✓		(32)
Tea	Others	↓	✓			(10)
Retinol (endogenous)	Others	↑	✓			(33)
Retinol (exogenous)	Others	↓	✓	✓		(34)
Vitamin D	Others	↓		✓	✓	(35, 36)
Vitamin C	Others	↓	✓			(37)
Magnesium	Others	↓	✓			(37)
Food additives (sweeteners, emulsifiers, etc.)	Others	↑	✓	✓		(21)
Maillard reaction products (e.g., advanced glycation end products)	Others	↑		✓		(21)

### 2.1 Carbohydrates: a metabolic Jekyll and Hyde involved in UC pathogenesis

Epidemiological studies on the association between sugar-sweetened beverage consumption and the UC risk have shown that individuals with a high intake of sugary and soft drinks have a higher risk of developing UC (38, 39). Furthermore, excess sucrose consumption has been linked to a 10% increase in the incidence of UC (40). A prospective cohort study additionally indicated that higher dietary sugar intake is associated with an increased risk of UC, although a significant correlation was not observed between total carbohydrate consumption and UC susceptibility (41). Excess sugar intake has been shown to upregulate the expression of soluble epoxide hydrolase, a key metabolic enzyme in the gut, and to promote gut microbiota dysbiosis along with the expansion of pathogenic bacteria. These mechanisms are thought to contribute to metabolic-associated colonic inflammation induced by high-sugar diets.

Under physiological conditions, inflammatory cells primarily rely on lipid oxidation for energy. However, during the pathogenesis of UC, activated inflammatory cells shift toward a glycolytic-dependent metabolic phenotype to meet the high energy demands required for their activation, migration, and effector functions. This metabolic reprogramming from lipid oxidation to glycolysis supports rapid ATP generation and supplies essential biosynthetic precursors, facilitating cellular proliferation, differentiation, and the secretion of inflammatory mediators. Previous studies have consistently demonstrated that increased glycolytic flux is a hallmark of energy metabolism in inflammatory cells from UC patients (42).

### 2.2 Lipids: inflammatory rheostats in the colonic microenvironment

A large prospective cohort study conducted in adults in the UK revealed that excessive dietary intake of arachidonic acid is a significant risk factor for developing UC. Another meta-analysis demonstrated that long-term excessive dietary intake of fat, polyunsaturated fatty acids, omega-6 fatty acids, and cholesterol is associated with an increased risk of ulcerative colitis (18). Another epidemiological investigation also indicated that the chronic excessive intake of total fat, polyunsaturated fatty acids (PUFAs), and omega-6 fatty acids increases the risk of UC onset (43). Excessive fat intake is associated with increased free fatty acid levels in the gut, which may promote lipid peroxidation and compromise the intestinal mucosal barrier. Notably, a 6-month open-label, randomized, placebo-controlled trial revealed that increased dietary intake of n-3 polyunsaturated fatty acids (n-3 PUFAs) is associated with a reduced likelihood of developing UC (19, 35). The intake of n-3 PUFAs, specifically eicosapentaenoic acid (EPA) and docosahexaenoic acid (DHA), has been shown to lower the incidence of UC. n-3 PUFAs exert anti-inflammatory effects through multiple mechanisms and can promote the active resolution of inflammation (38). Therefore, increasing the consumption of n-3 PUFAs from oily fish may contribute to the prevention of UC in the general population (44). These findings reveal the dual nature of the impact of dietary fat on UC: its effects are highly dependent on the type of fatty acid.

A high-fat diet (HFD) has been shown to promote a proinflammatory adipose state and increase intestinal permeability in animal models, partly through alterations in the levels of tight junction proteins (occludin, ZO-1, and claudins) and the disruption of the epithelial damage/repair balance, which may facilitate the translocation of enteric pathogens and LPS. TLR4, which is activated by gut microbiota-derived LPS or dietary saturated fatty acids (SFAs), has been shown to activate NF-κB signaling and upregulate the expression of proinflammatory cytokines, which may further increase permeability, enable luminal antigens to access the lamina propria, and promote naïve T-cell differentiation and the secretion of TNF-α and IL-6 from macrophages, collectively contributing to inflammation and UC progression (20).

Concurrently, an HFD significantly reduces gut microbial diversity and the abundance of beneficial bacteria. This dysbiosis further disrupts the metabolic balance of fatty acids, bile acids, and tryptophan. Studies have indicated that HFD-induced gut dysbiosis and associated microbial metabolites may impair intestinal barrier function in experimental models through multiple mechanisms, including the promotion of intestinal epithelial cell apoptosis, inhibition of the secretory functions of goblet cells and Paneth cells, disruption of intercellular tight junctions, and induction of intestinal immune dysregulation. These pathological changes collectively contribute to intestinal mucosal barrier (IMB) dysfunction, which is considered a central event in the pathogenesis of HFD-associated UC (21).

In stark contrast, reduced dietary intake of n-3 PUFAs is also associated with an increased risk of UC. n-3 PUFAs have been reported to exert protective effects, partially through competitive inhibition of arachidonic acid metabolic pathways, which reduces the production of proinflammatory mediators such as prostaglandins and leukotrienes. Additionally, they have been shown to modulate the PPARγ and NF-κB signaling pathways and to suppress vascular cell adhesion and migration, angiogenesis, and adaptive immune responses.

The effects of dietary lipids on UC are complex and may depend on the type, source, and balance of fatty acids rather than total fat intake alone. Differences in dietary assessments, population characteristics, and the gut microbiota composition may partly explain the inconsistent findings among studies.

### 2.3 Proteins and amino acids: the central hub of gut mucosal signaling

High animal protein intake is a risk factor for UC. A meta-analysis of fourteen epidemiological studies revealed a significant association between meat (particularly red meat) consumption and an increased UC risk (22); another study showed a positive correlation between meat intake and UC relapse, whereas legume consumption over one year was inversely associated with relapse, suggesting a protective role for plant-based proteins (45, 46). Excess protein, especially of animal origin, reaches the colon undigested and is fermented by the gut microbiota, generating toxic byproducts such as hydrogen sulfide that have been shown to damage colonic epithelial cells in experimental settings.

Fat mass and obesity-associated protein (FTO) downregulates ceramide synthase 6 (CerS6) expression via an m6A-dependent mechanism in murine models, leading to abnormal sphingosine-1-phosphate (S1P) accumulation in intestinal epithelial cells (IECs) and potentially exacerbating UC progression (23). ID2, a widely expressed ID protein family member, modulates cellular processes by binding to target proteins and inhibiting their DNA-binding capacity; ID2 overexpression suppresses intestinal epithelial cell apoptosis, preserves mucosal barrier function in experimental models, and ameliorates UC by alleviating clinical symptoms, reducing the colonic levels of proinflammatory cytokines, enhancing barrier integrity, and attenuating immune cell infiltration. Exogenous ID2 supplementation may represent a novel therapeutic strategy for UC (24).

Tryptophan (TRP), an essential amino acid, is metabolized through three primary pathways: the kynurenine (Kyn), serotonin (5-HT), and indole pathways. These pathways convert TRP into various bioactive metabolites with immunomodulatory properties, which contribute to the maintenance of immune homeostasis. The dysregulation of TRP and its metabolites is associated with an immune imbalance, intestinal inflammation, the disruption of the epithelial barrier, and the activation of inflammatory pathways in individuals with UC. This metabolic imbalance may contribute to chronic inflammation and disease progression through the stimulation of immune cell infiltration and cytokine release (25).

Key branching enzymes in the kynurenine pathway—kynurenine 3-monooxygenase (KMO) and kynureninase (KYNU)—play important roles in the pathogenesis of UC. Studies have shown the significant dysregulation of tryptophan metabolism in UC patients, which is characterized by abnormal upregulation of KMO and KYNU expression. This upregulation is positively correlated with the levels of the proinflammatory cytokines TNF-α and IL-1β, suggesting that the inflammatory microenvironment may contribute to UC progression through the modulation of the kynurenine metabolic pathway (26).

Taurine, a sulfur-containing β-amino acid with important physiological functions, is biosynthesized primarily via the metabolic conversion of cysteine catalyzed by cysteine sulfinic acid decarboxylase (CSAD) and can also be obtained through dietary intake via taurine transporters. Research has indicated that taurine exerts protective effects on the intestinal mucosa in experimental models through multiple mechanisms, including the maintenance of intestinal epithelial integrity, downregulation of proinflammatory cytokine expression, and anti-inflammatory activity mediated by its abundance in immune cells (27).

Aspartate (Asp), a major energy source for the intestinal mucosa, maintains barrier integrity and regulates nucleotide, protein, lipid, and energy metabolism. Aspartate supplementation was shown to upregulate RIPK1 and RIPK3 expression, downregulate MLKL expression, and increase the abundance of beneficial bacteria (*Bacteroides*, *Lactobacillus*, and *Alistipes*) in murine models, suggesting a potential role in promoting colonic repair and facilitating UC recovery through the enhancement of immune/antioxidant functions, the modulation of RIPK signaling, and the reshaping of the gut microbiota (28). The protective effects of glutamine on UC are reflected in two parallel dimensions: epithelial barrier maintenance and immune regulation. On the one hand, glutamine supports intestinal epithelial cell proliferation and barrier repair through multiple signaling pathways. After it is hydrolyzed into glutamate by glutaminase, glutamine enters the tricarboxylic acid cycle and serves as a major energy source for intestinal epithelial cells. It also activates mTORC1 signaling to promote protein synthesis and cell proliferation. In addition, glutamine promotes intestinal epithelial cell proliferation via the ERK/MAPK pathway, upregulates tight junction protein expression, and thus improves intestinal barrier function and maintains intestinal permeability. In terms of maintaining barrier integrity, glutamine enhances the physical function of the epithelial barrier by upregulating the expression of tight junction proteins such as occludin and claudin-1. It also reinforces the chemical defense of the mucosal barrier by promoting the synthesis and secretion of the mucin MUC2. In terms of antioxidant defenses, glutamine acts as a rate-limiting substrate for glutathione synthesis, which is catalyzed by γ-glutamylcysteine ligase to generate glutathione, increasing the antioxidant capacity of intestinal epithelial cells and reducing oxidative stress-induced epithelial damage. On the other hand, glutamine exerts immunomodulatory effects through multiple molecular mechanisms. It effectively inhibits the NF-κB/MAPK/PI3K signaling cascade by blocking IκBα phosphorylation and p65 nuclear translocation upon LPS or TNF-α stimulation, thus reducing the transcription of proinflammatory mediators. Moreover, glutamine accelerates MAPK dephosphorylation by inducing MKP-1 expression, providing negative feedback to suppress sustained inflammatory signaling. Glutamine also reduces the expression of NOS2 and COX-2, promotes IL-10 secretion and anti-inflammatory macrophage polarization, and downregulates the expression of the proinflammatory cytokine IL-6. At the epigenetic level, glutamine upregulates NCOA3 expression by inhibiting WTAP-mediated m6A methylation of the NCOA3 mRNA, further alleviating inflammation (47–49).

Notably, approximately 6–18 g of protein substrates reach the colon daily for fermentation and are mainly derived from undigested dietary proteins and partly from endogenous secretion; the gut microbiota (e.g., *Escherichia coli*, *Clostridium*, *Bacteroides*, and *Klebsiella pneumoniae*) ferment these proteins to produce toxic metabolites, including H_2_S, which may disrupt cellular membrane synthesis, butyrate β-oxidation, mitochondrial respiration, and immune function, collectively contributing to epithelial barrier disruption and UC pathogenesis (50).

### 2.4 Dietary fiber and metabolites: the guardians of mucosal homeostasis

Meta-analyses have shown that the regular consumption of fiber-rich fruits and vegetables reduces the incidence of UC, and a Mediterranean diet, which is high in dietary fiber, is also considered an effective dietary pattern for decreasing intestinal inflammation and preventing UC (29). This protective effect is likely attributed to the fermentation of soluble dietary fiber by the gut microbiota to produce SCFAs such as butyrate, which exert anti-inflammatory effects.

In animal models, dietary fiber depletion has been shown to shift the colonic environment toward a proinflammatory state, increase neutrophil recruitment, and exacerbate UC development. Conversely, SCFAs produced by the microbial fermentation of a fiber-rich diet have been reported to suppress inflammation, partly by improving epithelial barrier function and modulating relevant receptors (51). Among these SCFAs, acetate is the most abundant in the colonic lumen and circulation. It can regulate leukocyte recruitment, induce the generation of regulatory T cells, and enhance their function. It has also been hypothesized that neutrophils are retained in the circulation through CXCL2–CXCR interactions and may contribute to the prevention of UC onset. Notably, the protective mechanisms of dietary fiber extend beyond SCFA production through fermentation. For example, lignin, an insoluble and poorly fermentable fiber component, can protect the intestinal epithelium from ferroptosis by binding to GPR37 and activating the ERK–NRF2–GPX4 signaling pathway (30). In experimental models, quinoa bran soluble dietary fiber (QBSDF) increases acetate and butyrate concentrations, downregulates TNF-α and IL-1β at both the transcript and protein levels, upregulates tight junction protein expression, and remodels the gut microbiota composition. It increases microbial diversity and the abundance of Firmicutes while reducing the abundance of Bacteroidetes and pathogenic *Helicobacter*, suggesting a potential role in inhibiting colonocyte apoptosis (31). Similarly, high-purity insoluble dietary fiber (HPIDF) intake was shown to modulate SCFA biosynthesis in animal studies by regulating the proliferation of key bacteria (including *Shigella*, *Lactobacillus*, and Prevotellaceae) in the PWY-2941 and AEROBACTINSYN-PWY metabolic pathways, which may contribute to the alleviation of UC symptoms (32).

In summary, dietary fiber is associated with reduced risks of the initiation and progression of UC, potentially through multiple mechanisms. However, due to the inherent differences among fiber sources in the ratio of fermentable to nonfermentable components, chemical structure, and fermentation kinetics, as well as variations in the administration route, disease stage, and epithelial metabolic capacity, intervention studies using different fiber components often exhibit heterogeneity in the effect sizes and dominant mechanisms. This variability further underscores the need to clarify fiber structure–function relationships and subsequently screen for optimal fiber combinations.

### 2.5 Other dietary factors and UC

Dietary additives can also influence the occurrence of UC by modulating the gut microbiota and metabolic pathways.

In terms of harmful factors, food additives and byproducts generated during food processing have attracted considerable attention. Sweeteners and emulsifiers that are widely present in high-fat diets, along with Maillard reaction products (such as advanced glycation end products) formed during thermal processing, can impair intestinal mucosal barrier function, thus promoting the onset and exacerbation of UC (21). Epidemiological evidence supports this association, with studies demonstrating a positive correlation between the consumption of sweetener-containing foods and the UC risk, suggesting a close link between the intake of ultra-processed foods and intestinal health (52, 53).

With respect to protective factors, tea-drinking habits are significantly associated with a reduced risk of UC, which may be attributable to the antioxidant and anti-inflammatory properties of tea-derived polyphenols that scavenge free radicals and protect cells from oxidative damage (39). Diets rich in fluids, magnesium, and vitamins C and D have also been shown to confer protection against UC (37). Among these factors, the role of vitamin D warrants particular attention. Vitamin D deficiency is prevalent in patients with IBD and is regarded as a potential dietary risk factor for increased disease susceptibility (35). Mechanistic studies have indicated that vitamin D alleviates UC through at least two pathways: it inhibits ferroptosis in intestinal epithelial cells by negatively regulating ACSL4, and it maintains mucosal barrier integrity while downregulating the expression of proinflammatory cytokines (36).

Retinol (vitamin A) and its metabolite retinoic acid play distinct roles in intestinal immunity. A substantial body of experimental evidence has demonstrated that exogenous supplementation with retinoic acid or its precursors has remarkable protective effects on colitis models through the induction of Foxp3^+^ regulatory T-cell differentiation, the suppression of Th1/Th17 effector programs, and the promotion of IL-10 secretion (34). However, these findings are derived predominantly from preventive interventions or *in vitro* studies and may not fully reflect the actual behavior of endogenous retinoic acid in patients with established active inflammation. A clinical study of UC patients offered a different perspective: retinoic acid levels are aberrantly elevated in the intestinal mucosa of patients with active disease, and its concentration is positively correlated with the levels of the proinflammatory cytokines IL-17 and IFN-γ but negatively correlated with the level of IL-10. In addition to classical CD4^+^ T cells, CD8^+^ T cells, MAIT cells, and γδ T cells have all been identified as important sources of IL-17 and IFN-γ, suggesting that in the active inflammatory microenvironment, endogenously elevated levels of retinoic acid may not execute an anti-inflammatory function but may instead drive the effector responses of multiple T-cell subsets. The molecular basis for this functional switch may lie in the ability of abundant proinflammatory signals, such as IL-6, within the inflammatory microenvironment to activate transcriptional networks, including STAT3, which engage in crosstalk with the retinoic acid–RARα signaling pathway to reprogram the target gene expression profile from anti-inflammatory tolerance toward a proinflammatory effector program (33). Thus, the role of retinoic acid in UC is intimately tied to the inflammatory microenvironment: the protective effects observed with exogenous supplementation and the proinflammatory consequences of endogenous dysregulation may coexist, positioning it as a bidirectional immune hub that is dynamically governed by the intensity of local inflammation.

Dietary–metabolic factors are considered important environmental contributors to UC, with metabolic dysfunction likely playing a central role in its pathogenesis. The chronic overconsumption of specific nutrients may induce systemic metabolic disturbances that may drive sustained metabolic reprogramming. Future research should elucidate how dietary–metabolic patterns systemically regulate the intestinal microenvironment in patients with UC. Targeting host–microbiota co-metabolic networks (e.g., short-chain fatty acid, bile acid, and tryptophan metabolism) may dynamically regulate gut nutrient availability and restore immune–microbial homeostasis. These strategies will provide targets for precision nutritional interventions (e.g., the timed supplementation of specific carbohydrates or proteins) and facilitate the development of predictive models that integrate multiomics data, which could link individual metabolic signatures to disease activity and the treatment response, ultimately enabling a closed-loop system of “dietary regulation–metabolic remodeling–prognostic prediction.”

## 3 The role of metabolic reprogramming in UC

We propose that the pathogenesis and progression of UC is essentially a vicious cycle triggered by dietary factors, amplified by metabolic dysregulation, and ultimately perpetuated by the reciprocal interplay among the epithelial barrier, immune responses, and the gut microbiota. Within this chain, metabolic reprogramming appears to serve as a pivotal hub that links upstream and downstream events: it represents a cellular response to the altered intestinal metabolic environment while simultaneously acting as a common origin for multiple downstream pathological consequences. Deprived of adequate energy substrates, intestinal epithelial cells face an “energy crisis” and are thought to shift from oxidative metabolism to glycolysis. Once initiated, this metabolic switch has been proposed to release disruptive signals in three directions simultaneously that compromise mucosal barrier integrity, create microenvironmental conditions that favor proinflammatory reprogramming of immune cells, and reshape the gut microbial community by altering the luminal chemical environment. These three directions are by no means independent of one another: barrier damage facilitates the translocation of luminal antigens, which further activates immune cells and promotes the release of inflammatory cytokines; the inflammatory microenvironment exacerbates mucosal hypoxia and oxidative stress, which in turn sustains and prolongs epithelial injury; and the reduction in SCFAs resulting from gut dysbiosis further depletes the epithelial energy supply. Through these interconnections, barrier disruption, immune activation, and microbial dysbiosis reinforce and amplify one another, ultimately forming a self-perpetuating pathological loop that may drive the persistent progression of UC (**Figure 3**).

**Figure 3:**
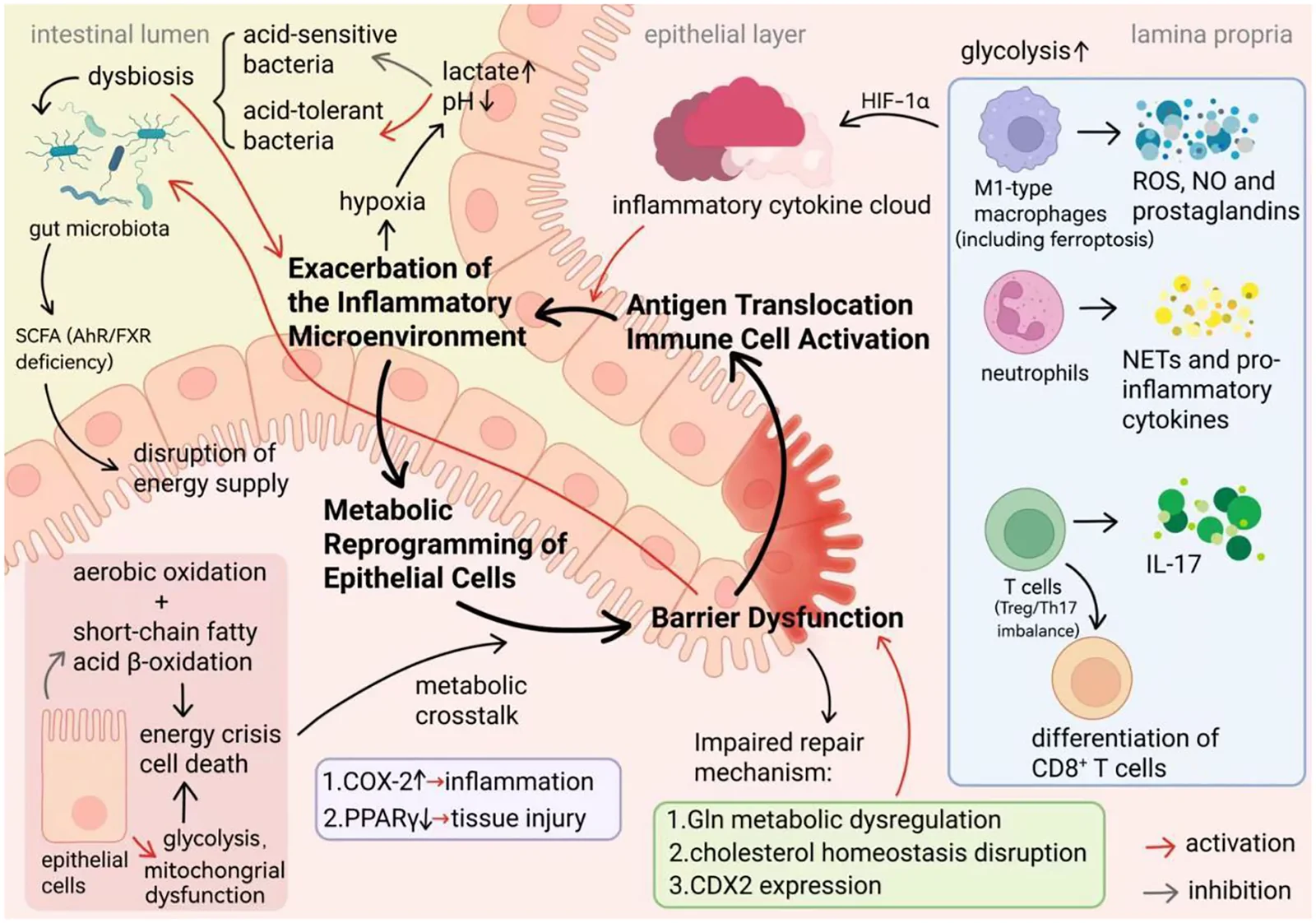
The central role of metabolic reprogramming in UC. In the gut lumen, dysbiosis, SCFA deficiency (AhR/FXR loss), and hypoxia acidify the environment and disrupt the epithelial energy supply. In the lamina propria, glycolysis-driven immune reprogramming includes M1 macrophage ferroptosis, NET release, and a Treg/Th17 imbalance (which promotes IL-17 production and CD8+ T-cell differentiation). These events, together with antigen translocation, result in epithelial barrier dysfunction and impaired repair. Some graphical elements in this figure were created using BioGDP.com (https://BioGDP.com) (16).

### 3.1 Initiation and core mechanisms of metabolic reprogramming in UC

The origin of this vicious cycle is thought to be the disruption of the intestinal metabolic environment by the consumption of a Western diet: insufficient dietary fiber leads to a sharp decrease in the production of short-chain fatty acids (SCFAs), particularly butyrate, on which colonic epithelial cells are highly dependent for energy through β-oxidation under normal physiological conditions. The resulting substrate deprivation is thought to induce an energy crisis in these cells. Concurrently, microcirculatory disturbances and immune cell infiltration during early inflammation create localized mucosal hypoxia. The convergence of hypoxia and substrate depletion is thought to drive the progression of metabolic reprogramming.

Hypoxia-inducible factor-1α (HIF-1α) plays a pivotal role in this metabolic reprogramming. The hypoxic microenvironment stabilizes HIF-1α and promotes its accumulation, which has been shown to rewire the cellular metabolic program. It upregulates the expression of glucose transporters and key glycolytic enzymes (such as HK2, PFKL, and PKM2), channeling carbon flux into glycolysis. Moreover, it induces pyruvate dehydrogenase kinase to block pyruvate entry into mitochondria, actively suppressing the tricarboxylic acid (TCA) cycle and oxidative phosphorylation. However, HIF-1α does not act in isolation, as proinflammatory signals transmitted via the NF-κB pathway form a positive feedback amplification loop with HIF-1α, while the downregulation of the expression of PPARγ, a key regulator of lipid metabolism, in the inflammatory milieu further suppresses the fatty acid β-oxidation pathway, leading to a sustained reduction in the expression of critical enzymes such as ACSL1 and ACADM. Through the synergistic actions of these signals, epithelial cells are thought to be locked into a metabolic mode characterized by increased glycolysis and restricted mitochondrial oxidation.

These transcriptional regulatory changes are manifested at the molecular execution level as the coordinated dysregulation of metabolic pathways, as described below. In the β-oxidation pathway, the expression of key enzymes such as ACSL1, ACADM, and ECHS1 is markedly reduced, which is associated with the impaired ability of the colonic epithelium to utilize butyrate (54, 55). The TCA cycle is suppressed because of an insufficient acetyl-CoA supply resulting from restricted glycolytic carbon flux and weakened fatty acid β-oxidation. Concurrently, reduced expression of UQCRC1, a key subunit of electron transport chain complex III, is associated with attenuated oxidative phosphorylation (56). Moreover, the downregulation of the expression of antioxidant proteins such as GST, GPX, ALDH2, and MSRA has been observed in individuals with UC, which superimposes oxidative stress upon the energy crisis. The regulation of glycolysis involves more complex alterations. Although HIF-1α promotes glycolysis at the transcriptional level, the expression of key enzymes such as HK1, GPI, PFKL, and ALDOA is significantly reduced at the protein level, suggesting that this process is not a simple hypoxic response but may involve posttranscriptional negative feedback mechanisms in the inflammatory environment. These changes collectively point to a central event, namely, mitochondrial dysfunction. The suppression of the TCA cycle and decreased activity of electron transport chain complexes severely impair oxidative phosphorylation, while damaged mitochondria, a major source of ROS, in turn exacerbate oxidative stress, creating a vicious subcycle of “metabolic reprogramming → mitochondrial damage → ROS accumulation → further metabolic disruption”. Thus, mitochondrial dysfunction is both a consequence of metabolic reprogramming and a key driver that perpetuates the energy crisis.

This metabolic shift precipitates an “energy crisis” in epithelial cells: insufficient ATP production compromises their ability to maintain tight junctions, secrete mucus, and support regenerative proliferation, which may render the mucosal barrier more vulnerable (see Section 2.2). The pathological significance of this shift extends far beyond a barrier impairment, as it simultaneously transmits disruptive signals in two additional directions. In the immune direction, once barrier function is compromised, luminal antigens are thought to flood into the lamina propria. Moreover, lactate accumulation and local acidification resulting from enhanced glycolysis have been suggested to provide favorable conditions for proinflammatory polarization and metabolic reprogramming of immune cells (see Section 2.3). In the microbial direction, increased glycolysis in epithelial cells increases luminal lactate levels and lowers the pH, whereas decreased mitochondrial oxidation reduces oxygen consumption and increases the luminal oxygen tension. These environmental changes selectively inhibit butyrate-producing obligate anaerobes and favor the proliferation of acid-tolerant bacteria, further contributing to gut dysbiosis and the depletion of the SCFA supply (see Section 2.4).

### 3.2 Metabolic reprogramming in intestinal epithelial cells: barrier dysfunction and damage repair

Intestinal epithelial cells are among the fastest-renewing cells in the body, and their normal metabolism is crucial for maintaining cellular function and intestinal tissue homeostasis (57). As early as 1980, W.E. Roediger proposed that UC is an energy metabolism disorder in the intestinal epithelium (58), highlighting the potential role of metabolic reprogramming in colonic epithelial damage and repair processes. Subsequent research has further suggested that metabolic alterations in epithelial cells are not only a consequence of intestinal inflammation but also a pathogenic factor that may contribute to its occurrence and development (59).

As the core manifestation of this pathogenic factor, metabolic disorders of colonic mucosal epithelial cells constitute the initiating step of the pathological process of UC. Under normal physiological conditions, these cells primarily rely on glucose aerobic oxidation and short-chain fatty acid β-oxidation for their energy supply. However, under pathological UC conditions, cells are thought to switch to anaerobic glycolysis due to the formation of a hypoxic mucosal microenvironment and the downregulated expression of key metabolic enzymes (54, 55, 60). This metabolic shift leads to an insufficient energy supply for epithelial cells, which may subsequently exhibit increased apoptosis and necrosis and ultimately compromise barrier integrity.

Metabolic disturbances culminate in core UC pathology: barrier dysfunction and impaired repair (**Figure 4**). Early defects in the intestinal epithelial barrier allow luminal antigens to flood the lamina propria, triggering a Th2-mediated immune response, immune cell infiltration, and proinflammatory cytokine release that may exacerbate inflammation. Goblet cell depletion severely compromises the MUC2 mucus layer (61). Additionally, inflammatory stimulation activates epithelial arachidonic acid metabolism, resulting in the production of ROS, leukotrienes, and prostaglandins that may drive cellular damage (62, 63).

**Figure 4:**
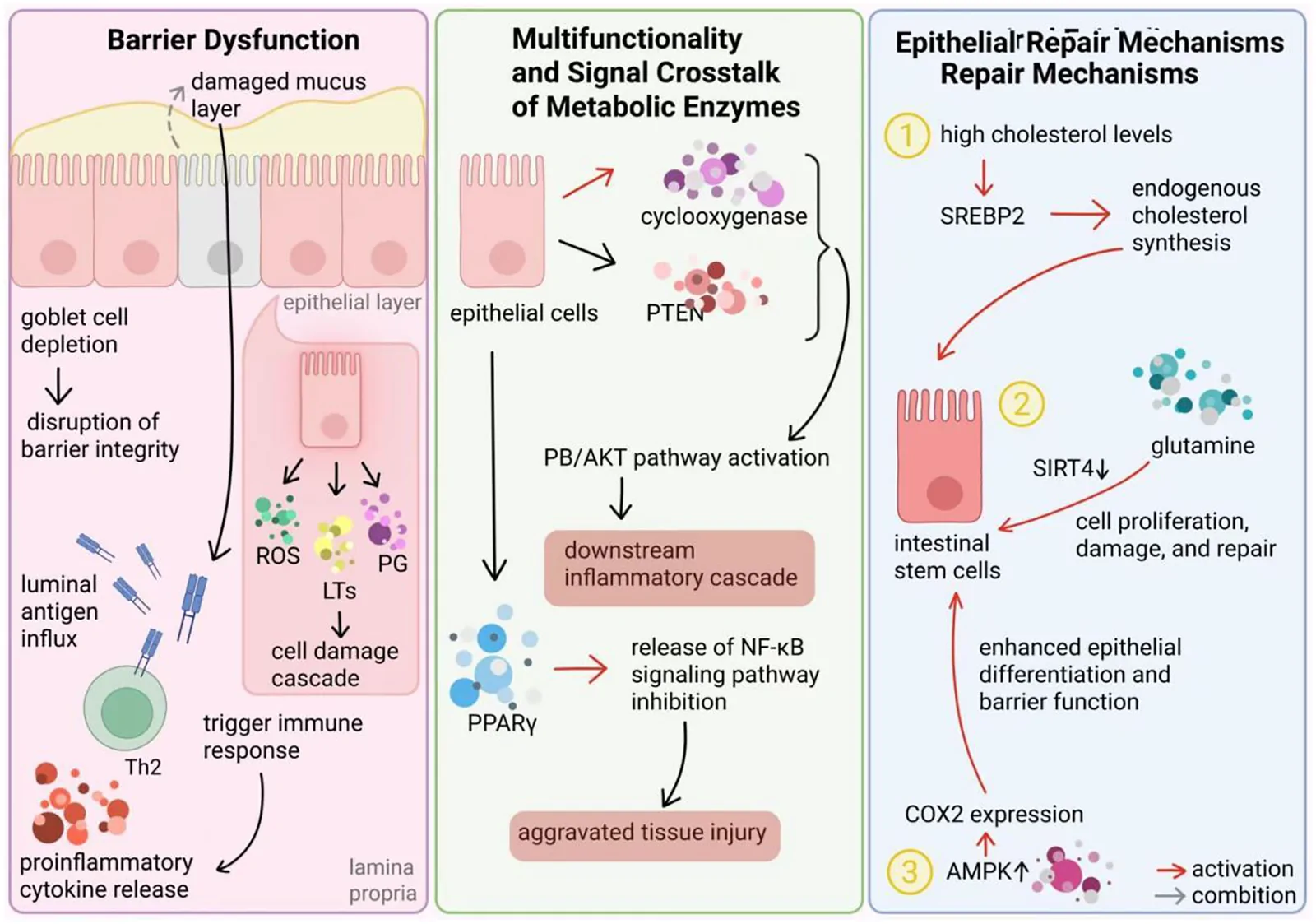
How a metabolic imbalance drives barrier dysfunction and impairs repair. Barrier damage involves goblet cell depletion, mucosal injury, Th2 activation, and ROS/LT/PG release. COX-2 upregulation activates PI3K/AKT signaling via PTEN inhibition, while PPARγ downregulation disinhibits NF-κB activity, aggravating injury. Impaired repair is driven by cholesterol-induced SREBP2 activation, SIRT4 downregulation-mediated glutamine utilization, and AMPK upregulation, which promotes differentiation/barrier function. Some graphical elements in this figure were created using BioGDP.com (https://BioGDP.com) (16).

In intestinal epithelial cells, certain metabolic enzymes possess multiple properties beyond their canonical metabolic functions, enabling their participation in other signaling pathways. Their abnormal expression induces both metabolic disturbances and altered crosstalk among signaling pathways, representing a potential mechanism by which metabolic reprogramming may trigger cascading effects in UC. For instance, arachidonic acid metabolism is activated upon inflammatory stimulation, and upregulated COX-2 expression suppresses PTEN, alleviating the negative regulation of PI3K/AKT activity and triggering downstream inflammation. Similarly, the lipid/glucose metabolism regulator PPARγ inhibits NF-κB activation, and inflammation-driven PPARγ downregulation disinhibits NF-κB activity, which may aggravate tissue damage (64, 65).

In addition to directly participating in inflammation and barrier destruction, metabolic reprogramming may hinder tissue healing by affecting epithelial repair mechanisms. Following intestinal injury, stem cells need to rapidly take up glutamine to support nucleotide synthesis. Recent research has shown that the deacetylase SIRT4 acts as a metabolic switch in the regulation of this process: its downregulation under stress conditions increases glutamine uptake and promotes cell proliferation and damage repair through *de novo* nucleotide synthesis (66). Epithelial repair also relies on cholesterol metabolic homeostasis. High cholesterol levels have been shown to promote endogenous cholesterol synthesis by activating SREBP2 in experimental models, thus accelerating stem cell proliferation and potentially alleviating UC (67). The core energy metabolism regulator AMPK also plays a key role in maintaining barrier function. Animal experiments have shown that AMPK deficiency exacerbates colitis, while its upregulation can enhance epithelial differentiation and barrier function by promoting CDX2 expression (68). This protective effect is mediated in part by the negative regulation of mTOR by AMPK. AMPK phosphorylation inhibits mTOR activity, relieving the mTOR-mediated blockade of autophagy and allowing the clearance of damaged tight junction proteins and mitochondria, thus preserving epithelial barrier integrity (69). AMPK also maintains intracellular NAD^+^ levels by regulating the NAMPT-mediated NAD^+^ salvage pathway, in turn ensuring normal glycolytic and oxidative phosphorylation functions. When AMPK activity decreases, the disruption in NAD^+^ metabolism further exacerbates mitochondrial dysfunction and the epithelial energy crisis (70).

### 3.3 Immunometabolic reprogramming: shaping the inflammatory response

The immunopathological process of UC involves dysfunctions in multiple components of both the innate and adaptive immune systems, with metabolic reprogramming considered a core mechanism driving this process (71). In the inflammatory microenvironment of UC, immune cells generally shift their energy metabolism from oxidative phosphorylation to glycolysis to meet the high energy demands during activation and function. This metabolic shift has been reported to regulate the activation status, functional polarization, and inflammatory cytokine secretion of various key immune cells (such as macrophages, neutrophils, and T cells), thus shaping the overall immune response and sustaining chronic inflammation (72) (**Table 2**; **Figure 5**).

**Table 2:** Immune cell metabolic reprogramming in UC.

Immune cell type	Metabolic shift	Key regulators/metabolites	Functional consequence in UC	References
Macrophages (M1)	Glycolysis ↑	GLUT1, HK2, PKM2, PFKFB3, succinate, citrate	Proinflammatory mediator (NO, ROS, and prostaglandins) production; M1 polarization	(73, 74)
Macrophages (M2)	Oxidative phosphorylation ↑	IL-10, glutamine (enhances the TCA cycle	anti-inflammatory phenotype; M2 polarization	(75, 76)
Neutrophils	Glycolysis (primary), gluconeogenesis (under low-glucose conditions)	HIF-1α (by LPS/TNF-α), glutamine (gluconeogenic substrate)	Rapid ATP generation; NET release (promoted by cholesterol depletion and sphingomyelin degradation) → tissue injury	(77–80)
CD4^+^T-cells (Th17)	Aerobic glycolysis ↑	Lactate (via SLC5A12)	Migration inhibition; Th17 differentiation; IL-17 production	(81, 82)
CD8^+^T-cells (Effector)	Aerobic glycolysis ↑	Lactate (via SLC16A1)	Inhibition of motility and cytotoxicity; terminal differentiation	(81, 82)
CD8^+^T-cells (Memory)	FAO ↑	SCFAs (↑ fatty acid uptake/oxidation); glycolysis inhibition	Long-lived memory cell formation	(82, 83)

**Figure 5:**
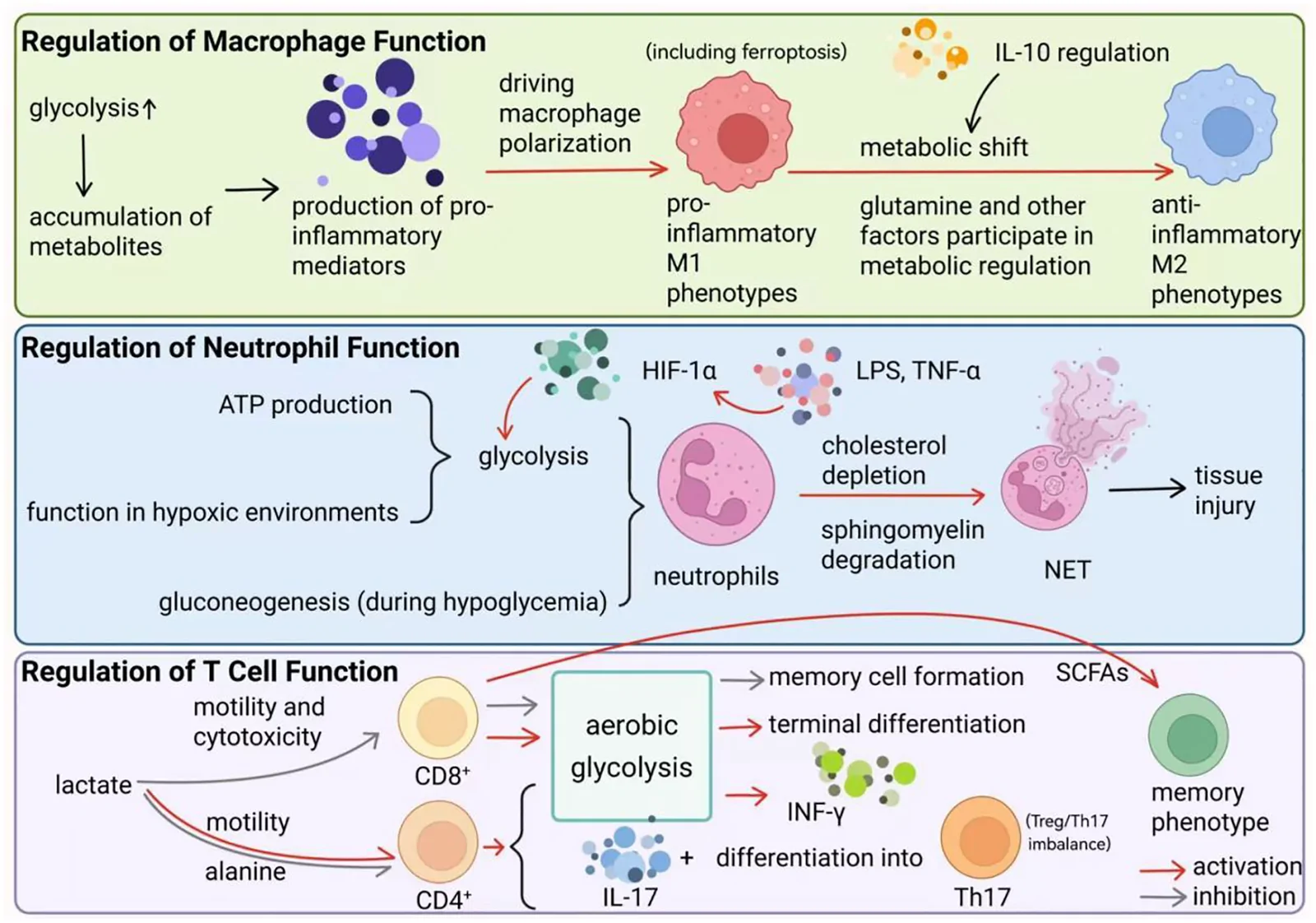
Immune cell metabolic reprogramming drives inflammation in UC. (Upper panel) In macrophages, increased glycolysis drives M1 polarization via metabolite accumulation and proinflammatory mediator release, while IL-10 and glutamine promote M2 polarization. Notably, metabolic dysregulation triggers ferroptosis in M1 macrophages. (Middle panel) In neutrophils, glycolysis provides ATP and supports cellular function under hypoxic conditions (HIF-1α). Cholesterol depletion and sphingomyelin degradation trigger NET formation, leading to tissue injury. (Lower panel) In T cells, lactate and alanine regulate the motility and cytotoxicity of CD8^+^ and CD4^+^ T cells. Aerobic glycolysis drives terminal differentiation and Th17 polarization (IL-17/IFN-γ) along with a Treg/Th17 imbalance, whereas SCFAs promote the generation of memory T cells. Some graphical elements in this figure were created using BioGDP.com (https://BioGDP.com) (16).

#### 3.3.1 Metabolic reprogramming regulates macrophage function

Macrophages are key regulators of intestinal immune homeostasis and play a critical role in the progression of UC (84). The proinflammatory phenotype of macrophages in the inflammatory microenvironment is regulated by their glycolytic metabolic activity (73). Studies have shown that macrophages undergo metabolic reprogramming via the Warburg effect, shifting from oxidative phosphorylation to glycolysis, and subsequently polarize into the proinflammatory M1 phenotype (60). Notably, the traditional M1/M2 binary model can no longer fully account for the functional diversity of macrophages in UC. Single-cell RNA sequencing studies have revealed the presence of nonclassically polarized subsets, such as S100A8^+^ and IL1B^+^ macrophages, in inflamed UC tissues. These subsets exhibit low signature scores for both M1 and M2 markers, suggesting that their activation follows a noncanonical polarization trajectory. Among these markers, S100A4 has been identified as a core regulatory node that drives proinflammatory macrophage polarization through interactions with RAGE and TLR4 to activate the NF-κB and MAPK pathways and subsequently induce iNOS and TNF-α expression, whereas the inhibition of S100A4 restores M2 marker levels and ameliorates colonic inflammation (85). HIF-1α acts as a key regulator of macrophage function in this process. Upon binding to HIF-1β, it targets hypoxia response elements to directly upregulate the transcription of glycolytic genes while also promoting macrophage survival and proliferation in the inflammatory environment. Elevated HIF-1α expression often indicates worsening inflammation (69). Increased expression of glycolytic proteins, such as GLUT1, HK2, PKM2, and PFKFB3, in macrophages promotes glycolytic flux, leading to the accumulation of metabolites such as succinate and citrate. This metabolic shift facilitates the production of proinflammatory mediators, including nitric oxide (NO), ROS, and prostaglandins, promoting macrophage polarization toward the proinflammatory M1 phenotype (74).

The application of single-cell transcriptomics has further revealed specific molecular features of macrophages. Studies based on single-cell RNA sequencing have identified four genes (HIF1A, S100A8, CYBB, and GLS) that are closely associated with ferroptosis in UC. Among these genes, the expression of CYBB, which encodes the core catalytic subunit of NADPH oxidase, is significantly upregulated in macrophages from individuals with UC. Its aberrant expression leads to ROS accumulation and dysregulated iron homeostasis, ultimately triggering ferroptosis, suggesting that the CYBB–ROS–ferroptosis module may represent one of the key mechanisms underlying proinflammatory macrophage dysregulation in individuals with UC (86). From a metabolic perspective, a close biochemical link exists between glycolytic reprogramming and ferroptosis in macrophages. During M1 polarization, increased glycolysis is accompanied by the relative suppression of the pentose phosphate pathway, resulting in insufficient NADPH regeneration and consequently impaired glutathione (GSH) synthesis. GSH is an indispensable cofactor for glutathione peroxidase 4 (GPX4), which is the only known selenoprotein capable of directly reducing lipid peroxides on biological membranes. Once GSH is depleted, GPX4 can no longer effectively eliminate lipid peroxides generated by ROS attack on membrane phospholipids, leading to their persistent accumulation and ultimately triggering ferroptosis. Thus, metabolic reprogramming in macrophages, particularly the imbalance between glycolysis and the pentose phosphate pathway, creates permissive metabolic conditions for ferroptosis through the GSH–GPX4 axis, while aberrant CYBB upregulation further exacerbates ROS accumulation against this backdrop of compromised antioxidant defenses, collectively driving ferroptosis execution through increased oxidative damage and weakened antioxidant protection (87).

Furthermore, the application of spatial multiomics technologies has provided additional support for metabolic reprogramming in macrophages. A study integrating transcriptomic, spatial transcriptomic, and proteomic analyses revealed a marked increase in SPP1-high macrophage subsets at sites of inflammation, indicating not only that metabolic reprogramming-driven macrophage polarization is unevenly distributed but also that it is distinctly enriched at inflammatory foci, providing direct evidence for the tissue specificity of macrophage phenotypes (88).

Interleukin-10 (IL-10) can shift macrophage metabolism from glycolysis to oxidative phosphorylation, resulting in a switch in the macrophage phenotype from proinflammatory to anti-inflammatory (75). This bidirectional metabolic–phenotypic plasticity constitutes the biochemical basis of the macrophage functional continuum. Additionally, glutamine serves as another key immunometabolic regulator in macrophages by enhancing the tricarboxylic acid (TCA) cycle and oxidative phosphorylation, which supports M2 polarization. Other metabolic pathways, including arginine metabolism, tryptophan metabolism, and fatty acid metabolism, have also been implicated in the regulation of macrophage-mediated immune responses (76).

#### 3.3.2 Metabolic reprogramming regulates neutrophil function

As a key component of the innate immune system, neutrophils contribute to the regulation of intestinal inflammation through phagocytosis and the production of inflammatory mediators (89). Glycolysis has long been recognized as a central pathway that governs neutrophil function. However, emerging evidence indicates that neutrophil activity is not solely dependent on glycolysis and that its interplay with metabolic reprogramming is considerably more extensive and complex than previously understood (90).

Glycolysis serves as the primary energy source for neutrophils, irrespective of oxygen availability in their microenvironment (77). This unique metabolic preference enables rapid adenosine triphosphate (ATP) generation, supporting swift responses during antibacterial and defense processes, and allows neutrophils to function effectively even under hypoxic or otherwise challenging conditions. In UC, excessive or dysregulated neutrophil activity is a major contributor to inflammatory progression and has been linked to hyperactivated glycolysis (78). Multiple stimuli, including lipopolysaccharide (LPS) and TNF-α, increase the expression of HIF-1α in neutrophils, and HIF-1α signaling has been shown to increase glycolysis.

Under low-glucose conditions, neutrophils can also use gluconeogenesis to convert nonglucose substrates into glucose and glycogen and subsequently sustain neutrophil-mediated responses in tissues. Glutamine has been identified as a critical nutrient source for neutrophil gluconeogenesis. Upon LPS stimulation, the utilization of glutamine by neutrophils increases to meet the energy demands (79). Furthermore, lipid metabolism plays a significant role in modulating neutrophil function. The availability of extracellular lipids influences human neutrophil survival and activity. Neutrophils deploy neutrophil extracellular traps (NETs) to capture and eliminate bacteria; however, under pathological UC conditions, excessive NET formation can exacerbate tissue damage. Studies have shown that both cholesterol depletion and sphingomyelin degradation promote NET release, which may aggravate inflammatory injury (80).

#### 3.3.3 Metabolic reprogramming modulates T-cell function

During inflammatory responses, T-cell activation is constrained by cellular metabolism, which supplies the energy and biomolecules necessary for T-cell growth, proliferation, and effector functions (91). Lactate, the end product of glycolysis, accumulates in the microenvironment under inflammatory and hypoxic conditions. Extracellular lactate has been shown to modulate T-cell function via specific lactate transporters on the cell surface (92). Lactate enters CD8^+^ T cells through solute carrier family 16 member 1 (SLC16A1), ultimately inhibiting their motility and cytotoxic activity. Similarly, sodium lactate is transported into CD4^+^ T cells via solute carrier family 5 member 12 (SLC5A12), impairing CD4^+^ T-cell migration. Furthermore, sodium lactate promotes the differentiation of CD4^+^ helper T cells into the Th17 subset, leading to increased production of the proinflammatory cytokine interleukin-17 (IL-17) (81, 93).

Upon activation, both CD4^+^ and CD8^+^ T cells engage aerobic glycolysis, but the extent and significance of this metabolic program differ between the two cell types (82, 94). In CD4^+^ T cells, glycolytic activity directly governs functional polarization: high glycolytic flux drives differentiation toward proinflammatory subsets such as Th17 cells and promotes IFNG gene expression and IFN-γ production, whereas decreased glycolysis creates metabolic conditions permissive for Treg differentiation. In this sense, the glycolytic pathway functions as a key immunometabolic checkpoint in T cells, with its activity determining whether T cells acquire a proinflammatory effector phenotype or maintain immune tolerance. In addition, the abundant ATP generated by glycolysis fuels PI3K signaling, further enhancing effector T-cell function (95). In CD8^+^ T cells, the intensity of glycolysis predominantly governs the balance between effector differentiation and memory formation. Increased glycolysis drives these cells toward a terminally differentiated effector state, increasing their rapid cytotoxic capacity, whereas the suppression of glycolysis favors the long-term survival of memory CD8^+^ T cells (96).

In contrast to the glycolytic dependence of Th17 cells, the immunosuppressive function of Treg cells relies upon a metabolic program dominated by mitochondrial oxidative phosphorylation and fatty acid oxidation. Under homeostatic conditions, Treg cells restrain the excessive activation of effector T cells by secreting IL-10 and TGF-β, with the STAT3 pathway activated by IL-10 receptor engagement playing a central role in suppressing Th1- and Th17-mediated inflammatory responses. In the inflammatory microenvironment of UC, however, although Treg cells may undergo compensatory expansion, both their capacity to secrete IL-10 and TGF-β and the stability of Foxp3 expression are diminished, rendering their immunosuppressive function insufficient to counteract the ongoing inflammatory response. More critically, the proinflammatory milieu can induce Foxp3^+^ Treg cells to ectopically express RORγt, endowing them with the potential to transdifferentiate into Th17 cells (97). Thus, the balance between Th17 and Treg cells is not merely a contest of cell numbers but a dynamic process that is jointly shaped by metabolic regulation and transcriptional networks, as the shift between glycolysis and oxidative phosphorylation and the antagonism between RORγt and Foxp3 fundamentally determine whether T-cell immune responses are driven toward inflammatory amplification or immune tolerance. Notably, this metabolic program characterized by fatty acid oxidation is not unique to Treg cells; it is similarly adopted by memory T cells, albeit for a fundamentally different purpose.

Unlike Treg cells, which harness fatty acid oxidation to sustain immunosuppression, CD8^+^ T cells employ SCFA-driven fatty acid uptake and oxidation to fuel a distinct differentiation trajectory. SCFAs derived from the gut microbiota promote fatty acid uptake and oxidation in CD8^+^ T cells, remodeling their metabolic landscape and promoting their transition toward memory CD8^+^ T cells (83). Among these memory cells, a subset chooses to reside within the intestinal mucosa in the long term without recirculating into the bloodstream, and they are designated tissue-resident memory T cells (TRMs). This population, encompassing both CD4^+^ and CD8^+^ subsets, exhibits a striking duality in patients with UC. Under homeostatic conditions, CD103^+^ CD8^+^ TRM cells maintain mucosal immune equilibrium, whereas in the inflammatory environment, CD4^+^ TRM cells accumulate in large numbers within the lamina propria and serve as a critical source of IL-17, and their abundance closely correlates with the UC relapse frequency. The concurrent enrichment of pathogenic CD8^+^ TRM subsets further amplifies tissue injury. This imbalance between protective residence and pathogenic expansion positions the targeting of aberrant TRM activation and retention as a direction of considerable interest for controlling UC relapse (98).

### 3.4 Gut microbiota and host metabolism: a dysfunctional partnership

A close bidirectional interaction has been observed between microbial metabolism and host metabolism in individuals with UC (**Figure 6**). The β-oxidation of SCFAs, particularly butyrate, serves as an important energy source for epithelial cells and is essential for maintaining mucosal homeostasis. In patients with UC, the reduced uptake and oxidation of SCFAs by epithelial cells may lead to an energy deficit that impairs mucosal homeostasis, epithelial self-renewal, and barrier function. Moreover, the metabolic reprogramming of epithelial cells can influence the intestinal microbial environment. When epithelial cells shift from oxidative phosphorylation and β-oxidation of SCFAs to glycolysis, their oxygen consumption decreases, resulting in increased oxygen availability in the intestinal lumen. This altered oxygen environment is unfavorable for obligate anaerobic bacteria and may further aggravate gut microbiota dysbiosis (77).

**Figure 6:**
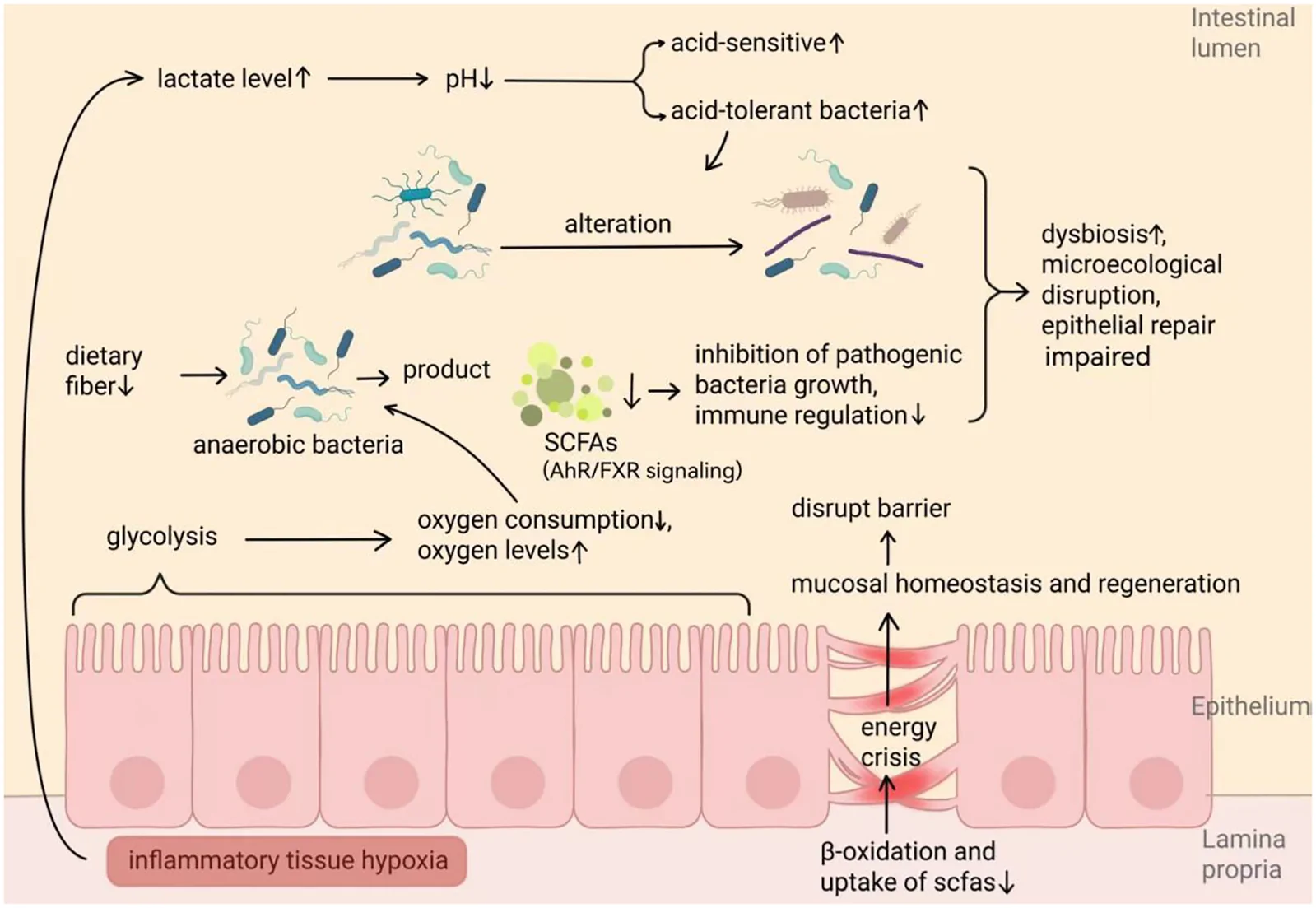
Microbiota–host metabolic crosstalk in UC. Reduced consumption of dietary fiber decreases SCFA production by anaerobic bacteria, whereas epithelial glycolysis reduces oxygen consumption and increases lactate levels, enriching acid-tolerant bacteria and driving dysbiosis. Diminished SCFA uptake and β-oxidation trigger epithelial energy crises, impairing mucosal homeostasis and barrier integrity, with SCFA-mediated AhR/FXR signaling deficiencies further compromising immune regulation. These reciprocal changes (reduced SCFA levels → barrier injury → hypoxia/glycolysis → altered microbiome → further SCFA loss) establish a bidirectional feedback loop that perpetuates UC progression. Some graphical elements in this figure were created using BioGDP.com (https://BioGDP.com) (16).

During the pathogenesis of UC, the gut microbiota undergoes significant metabolic reprogramming, as manifested by alterations in the microbial composition, metabolic pathways, and metabolite profiles. SCFAs are the primary metabolites produced by colonic anaerobic bacteria through the fermentation of dietary fiber. They inhibit the growth of pathogenic bacteria, such as *Escherichia coli* and *Clostridioides difficile*, regulate host immune responses, and contribute to the maintenance of intestinal homeostasis. However, due to changes in the gut microbiota composition and reduced dietary fiber intake in patients with UC, intestinal SCFA production is often decreased. The reduction in SCFA levels further weakens epithelial energy metabolism and barrier function, ultimately contributing to intestinal microecological disturbances and inflammation (99). The barrier-protective effects of SCFAs are mediated in part by the activation of the aryl hydrocarbon receptor (AhR). SCFAs such as butyrate can directly activate AhR signaling, promoting its nuclear translocation and upregulating IL-22 expression to consequently enhance the maintenance of tight junction proteins and intestinal barrier repair. In addition, ligands such as indole-3-carboxaldehyde, which is produced by the microbial metabolism of tryptophan, promote IL-22 secretion through AhR, helping to preserve mucosal barrier integrity and regulate the Th17/Treg balance (100, 101). Similarly, the bile acid receptors FXR and TGR5 are nuclear receptors activated by microbial metabolites that mediate a parallel barrier-protective pathway. The gut microbiota converts primary bile acids into secondary bile acids via bile salt hydrolases; the latter activate FXR to suppress NF-κB signaling and promote tight junction protein expression. In patients with UC, the loss of enzyme-producing bacteria such as *Lactobacillus* impairs secondary bile acid production, weakening FXR-dependent barrier protection (102). Thus, gut dysbiosis in patients with UC may simultaneously compromise the supply of both AhR and FXR ligands, disrupting the metabolic communication between the microbiota and the host at multiple receptor nodes.

Furthermore, the hypoxic and inflammatory microenvironment in patients with UC promotes glycolysis in intestinal epithelial cells and increases lactate accumulation in the intestinal lumen. Increased lactate levels decrease the intestinal pH, which inhibits the growth of acid-sensitive bacteria while promoting the proliferation of acid-tolerant bacteria. Thus, alterations in host metabolism, including reduced oxygen consumption and lactate accumulation, may reshape the ecological niche of the gut microbiota and contribute to changes in the microbial community structure. In turn, microbiota dysbiosis and decreased production of beneficial metabolites, such as SCFAs, further impair epithelial metabolism and repair. These changes form a bidirectional feedback loop between epithelial metabolic reprogramming and gut microbiota dysbiosis, which may promote the progression of UC (103, 104).

The bidirectional feedback model described above has gained preliminary support from human metabolomic and multiomics studies. One study, which was based on a randomized controlled trial, employed untargeted metabolomics and 16S amplicon sequencing to analyze fecal metabolites and the microbiota in patients with quiescent UC following the consumption of either a Mediterranean diet or a conventional diet. Patients with high adherence to the Mediterranean diet exhibited significant alterations in folate biosynthesis, sphingolipid biosynthesis, and steroid biosynthesis pathways, and individuals with a higher baseline abundance of fiber-degrading bacteria, such as *Bacteroides*, were more responsive to the dietary intervention (105). Another study integrated 16S rRNA gene sequencing, metagenomics, and metabolomics to profile adult and pediatric patients with UC and revealed marked age-related differences in the gut microbiota composition and metabolic signatures. Specifically, compared with adults, pediatric patients exhibited distinct microbial community structures and lower levels of short-chain fatty acids such as pentanoic acid and acetate, suggesting that microbial metabolic reprogramming is initiated early in UC and evolves dynamically with age and disease progression (106). Together, these two studies indicate that integrating human metabolomic profiles with microbiome data, while accounting for key host factors such as age and diet, represents an effective approach to elucidate the microbiota–host metabolic interplay in UC.

## 4 Targeting metabolism for UC management

### 4.1 Targeting key metabolic nodes: from small molecules to natural products

Modulating dysregulated metabolic pathways is emerging as a promising therapeutic strategy for UC (107). For instance, the JAK inhibitor tofacitinib controls the rate of glycolysis by regulating key glycolytic enzymes and has been clinically approved as a treatment for moderate-to-severe UC, effectively relieving symptoms and promoting mucosal healing (108). mTOR signaling also drives aerobic glycolysis; the mTOR inhibitor rapamycin has been shown to limit CD4^+^ T-cell infiltration in the colonic lamina propria and reduce IFN-γ production in animal models, consequently ameliorating colonic inflammation. Compared with rapamycin, the next-generation dual-target inhibitor AZD8055 simultaneously inhibits both mTORC1 and mTORC2, blocks mTOR signaling more effectively, and promotes Treg differentiation while suppressing Th1 and Th17 responses, further controlling colonic inflammation in experimental models (109–111).

Some small-molecule compounds have been shown to exert protective effects on UC. Aspartate (Asp) has been shown to improve colonic integrity and mitochondrial function; increase antioxidant enzyme activity; reduce apoptosis, necrosis, and inflammatory cytokine secretion; and remodel the gut microbiota in experimental models; its mechanism is associated with the inhibition of RIPK3/MLKL-mediated necroptosis through RIPK1 regulation (28). Narirutin (NR), a citrus flavonoid, has been shown to maintain intestinal barrier integrity by preventing the loss of tight junction proteins (claudin-3, occludin, and ZO-1) in experimental models. In addition, NR has been reported to suppress the TLR4-MAPK/NF-κB pathway and increase gut microbial diversity, as manifested by an increase in *Lactobacillus* abundance and decreases in *Bacteroides* and *Shigella* abundances. Notably, the anti-inflammatory effect of NR is largely dependent on the gut microbiota (112). Together, these findings suggest that Asp and NR have the potential to serve as prophylactic candidate treatments for UC.

Although targeting dysregulated metabolic pathways has shown therapeutic potential in UC, its clinical application remains challenging. Traditional orally administered drugs can achieve systemic bioavailability and improve clinical symptoms; however, their distribution is not restricted to the inflamed colon, which may lead to off-target effects and adverse systemic reactions. In contrast, biomimetic nanomedicine delivery systems may increase drug accumulation at inflammatory sites, reduce systemic exposure, and improve local therapeutic efficacy. Nevertheless, most of these delivery systems are still in the preclinical stage, and their stability, biosafety, and large-scale application require further investigation. Therefore, improving local targeting while reducing systemic adverse effects is important for metabolism-based therapy for patients with UC.

### 4.2 Advanced delivery systems: precision navigation to the inflamed gut

The rapid advancement of nanotechnology and biomaterials has enabled targeted drug delivery systems (TDDSs) to emerge as novel therapeutic strategies for UC that address the limitations described above. By increasing drug accumulation at the lesion site, reducing systemic exposure, and minimizing adverse effects, TDDSs have broad application prospects.

Macrophage-derived extracellular vesicles (MEVs) can adhere specifically to the inflamed vascular endothelium via ligand–receptor interactions, making them suitable for developing biomimetic drug delivery systems targeted to inflammatory regions. The excessive accumulation of ROS not only triggers pathological processes such as apoptosis and immune microenvironment disruption—which may promote the initiation and progression of UC—but also oxidizes cellular macromolecules, including proteins and lipids, and induces DNA damage under UC conditions. Researchers have developed a biomimetic nanodelivery system based on MEVs—a Pd@M nanoformulation composed of a palladium (Pd) cubic core and an MEV shell—to assess this mechanism. This system exhibits multienzyme-like activities and is capable of efficiently scavenging ROS, along with an excellent targeting ability and favorable biocompatibility. Experimental studies have confirmed that Pd@M modulates macrophage polarization by inhibiting the mTORC1–HIF-1α-mediated glycolytic pathway, which reduces neutrophil infiltration and recruitment, ameliorates colonic inflammation, remodels the immune microenvironment, and enhances mucosal barrier function in animal models; these findings suggest a potential strategy for colitis treatment (113).

Mannose and selenium (Se) possess potential anti-inflammatory and antioxidant properties. However, most lectin receptors exhibit relatively low affinity for free monosaccharide ligands, and many selenium species cause side effects within a very narrow dosage range. Selenium nanoparticles (M-SeNPs) were optimized and encapsulated within a colon-targeted hydrogel delivery system composed of sodium alginate (SA) and chitosan (CS) to overcome these limitations. After being taken up by intestinal epithelial cells (IECs), these nanoparticles upregulate the expression of selenium-dependent glutathione peroxidases (GPXs) to modulate immune responses in IECs. Moreover, CS/SA-embedded M-SeNPs (C/S-MSe) have been reported to inhibit the NF-κB pathway in the colon, alleviate oxidative stress and inflammation, stabilize mucosal homeostasis in intestinal epithelial cells, and improve colitis-associated symptoms in experimental models (114).

Despite their impressive targeting efficiency and therapeutic activity in preclinical models, the clinical translation of these biomimetic nanodelivery systems still faces considerable barriers. The existing evidence is derived almost exclusively from animal studies, and their long-term biosafety, immunogenicity, and batch-to-batch reproducibility must be verified in humans. Moreover, individual variations in the inflammatory microenvironment among UC patients may significantly affect the targeting efficiency of nanocarriers, but the factors governing this variability remain poorly understood. Therefore, advancing these systems from proof-of-concept to clinical application requires overcoming multiple hurdles, including safety evaluations, manufacturing standardization, and personalized adaptation.

### 4.3 Integrating dietary and microbiome-based interventions

Specific dietary components are associated with the occurrence and progression of UC, potentially through the regulation of metabolism. Adjusting the dietary structure has become an important component of disease management, as it may help correct metabolic abnormalities in UC patients. Clinical data indicate that dietary therapy can control clinical symptoms in UC patients and increase the endoscopic remission rate (115). The clinical practice for diet and nutritional therapy published by the American College of Gastroenterology recommends that UC patients without contraindications should follow a Mediterranean diet in their daily lives. This approach involves increasing the intake of various fresh fruits and vegetables, healthy fat sources of monounsaturated fatty acids, complex carbohydrates, and lean protein; reducing the consumption of ultra-processed foods and red meat; and controlling their dietary intake of sugar and salt (116).

Fecal microbiota transplantation (FMT) helps restore the levels of dysregulated gut microbiota-associated metabolites, including SCFAs and tryptophan-related metabolites, in patients by transplanting the microbiota from healthy donors into the patient’s body (117–119). Following FMT, the abundance of SCFAs in the patient’s intestine increases, which is associated with the differentiation of anti-inflammatory Treg cells and the alleviation of the UC inflammatory response (120). FMT can also increase the abundance of tryptophan metabolites such as kynurenine and serotonin in the gut, where they may exert anti-inflammatory effects by regulating T-cell-mediated immune responses (121).

The microbial metabolites H_2_S and NO have been implicated in the pathogenesis of UC, because they potentially damage colonic epithelial barrier function. Theoretically, regulating the production of these metabolites to reduce epithelial cell exposure may improve intestinal barrier function and alleviate mucosal inflammation. Previous research has indicated that dietary interventions can modulate colonic H_2_S production. Reducing the intake of sulfur-containing amino acids limits the supply of inorganic sulfur substrates, whereas increasing the intake of fermentable fiber has been shown to inhibit H_2_S production in experimental settings. In contrast, the mechanisms by which the diet regulates NO metabolism remain unclear. Therefore, although dietary strategies designed to regulate H_2_S production have potential therapeutic value, their clinical application requires further exploration and validation (53).

Although dietary interventions and FMT have shown some clinical potential in UC management, their adoption as standardized therapeutic strategies remains constrained by the level of evidence and the heterogeneity of existing protocols. The evidence supporting dietary interventions is derived primarily from observational studies and a small number of randomized controlled trials with limited sample sizes, which vary considerably in the dietary composition, intervention duration, and endpoint definitions. Consequently, a generalizable and standardized dietary protocol has yet to be established. FMT faces additional challenges, including inconsistent donor screening criteria, uncertainty regarding the optimal delivery route and dosing, and a lack of long-term safety data. More importantly, interindividual differences in the baseline gut microbiota composition may give rise to highly heterogeneous treatment responses, and the identification of potential responders remains a central unresolved issue. Therefore, at the current stage, dietary and microbiome-based interventions are best positioned as adjunct strategies within the comprehensive management of UC rather than as standalone therapies intended to replace established standard-of-care treatments (**Figure 7**; **Table 3**).

**Figure 7:**
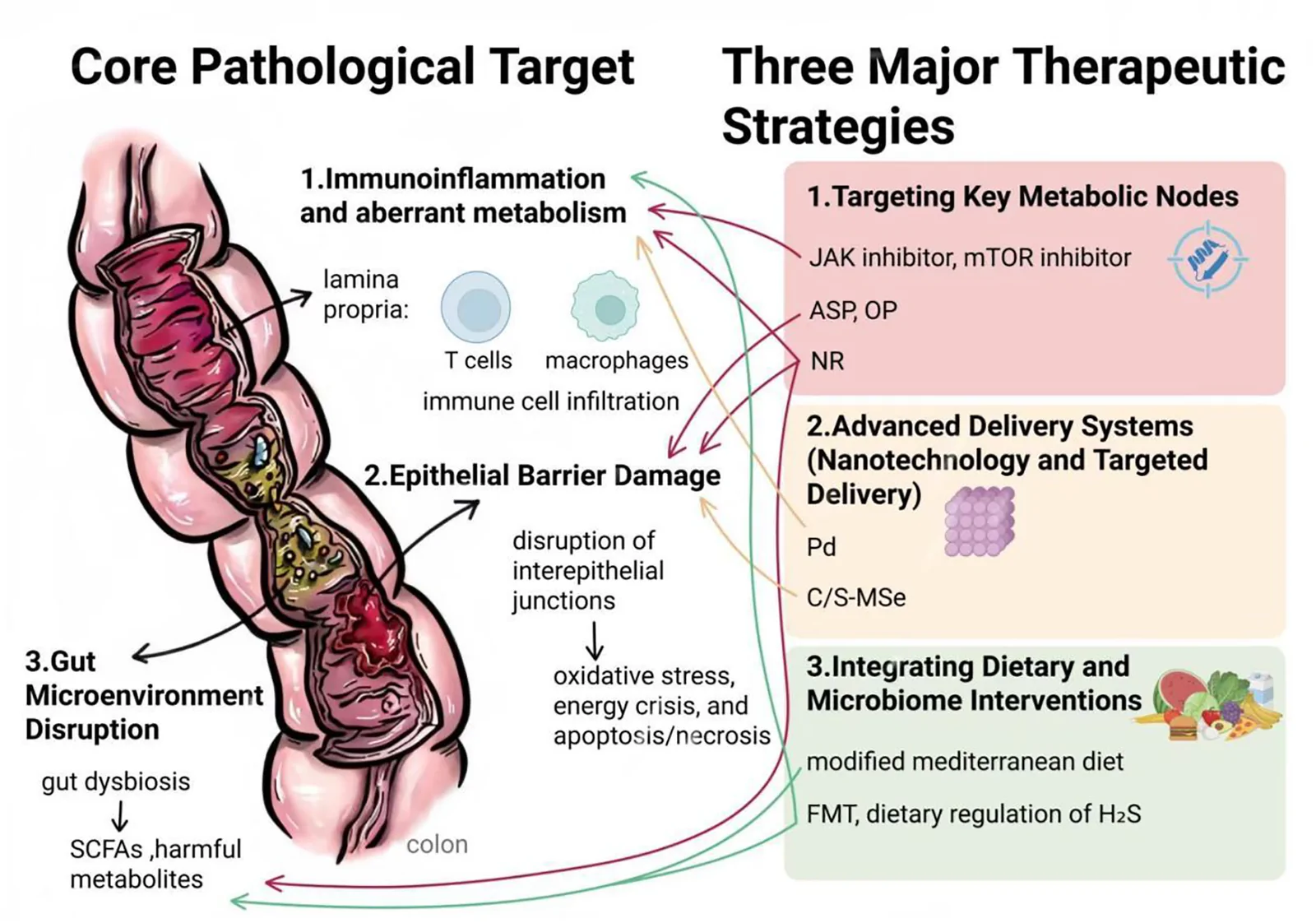
Strategies targeting metabolism for ulcerative colitis management. The core pathological targets include immunoinflammation with aberrant metabolism, epithelial barrier damage, and the disruption of the gut microenvironment. Strategies designed to address these issues by targeting key metabolic nodes involve the use of JAK/mTOR inhibitors and natural products (e.g., ASP and NR) to correct metabolic pathways and protect barrier function. Advanced delivery systems, such as Pd-based and C/S-MSe nanoplatforms, enable precise targeting of sites of inflammation with reduced systemic side effects. Integrated dietary and microbiome interventions, including a modified Mediterranean diet, FMT, and dietary regulation of H_2_S production, restore the levels of microbial metabolites such as SCFAs to improve mucosal homeostasis. Some graphical elements in this figure were created using BioGDP.com (https://BioGDP.com) (16).

**Table 3:** Evidence and translational barriers to metabolic therapies for UC.

Therapeutic strategy	Current evidence stage	Clinical applicability	Main limitations/translation barriers
JAK inhibitors (e.g., tofacitinib)	Some have been approved as treatments for moderate-to-severe UC	Partially available in clinical practice	Systemic, nonselective immunosuppression; risks of infection, thrombosis, and malignancy; lack of long-term real-world data
mTOR inhibitors (e.g., rapamycin and AZD8055)	Preclinical (animal models)	Not yet clinically applicable	Broad-spectrum immunosuppression; narrow therapeutic window; lack of cell-type specificity
Small-molecule compounds (e.g., aspartate and narirutin)	Preclinical (animal models)	Not yet clinically applicable	Lack of data from human clinical trials; low oral bioavailability; undefined pharmacokinetics and safety profiles
Biomimetic nanodelivery systems (e.g., Pd@M and C/S-MSe)	Preclinical (animal models)	Not yet clinically applicable	Unknown long-term biosafety; potential immunogenicity risks; batch-to-batch reproducibility; interindividual variability in the inflammatory microenvironment affecting targeting efficiency
Dietary interventions (e.g., the Mediterranean diet)	Mostly observational studies and a limited number of RCTs	Adjunct therapy	Lack of standardized intervention protocols; considerable heterogeneity across existing studies, with limited high-quality RCTs; challenges in long-term patient adherence
Fecal microbiota transplantation	Meta-analyses have confirmed short-term efficacy, but a standardized consensus has yet to be established	Adjunct therapy	Lack of standardization in donor screening, fecal preparation, and delivery procedures; limited data on long-term safety and microbial engraftment stability; highly heterogeneous treatment responses due to interindividual differences in the baseline gut microbiota composition

## 5 Critical considerations, knowledge gaps, and future directions

Current research on metabolic reprogramming in patients with UC still has considerable limitations. At the level of population-based evidence, most published epidemiological studies have employed prospective cohort designs. Although this approach strengthens causal inference by establishing temporality, the observational nature of these studies cannot fully exclude unknown or residual confounding. Dietary intake data are primarily collected through food frequency questionnaires or dietary history questionnaires, and recall bias and measurement error are inherent limitations of these tools. Rigorously designed randomized controlled trials that directly establish the causal effects of specific dietary components on UC incidence and the disease course are still lacking. Moreover, existing large-scale cohort studies have predominantly included European populations, and generalizing their findings to other ethnicities and regions is substantially constrained by differences in the genetic background, dietary culture, and baseline gut microbiota characteristics. Moreover, the extent to which interindividual heterogeneity in the microbiota composition and metabolic capacity influences the outcomes of dietary interventions remains unclear, and currently, neither reliable predictive biomarkers nor standardized evaluation criteria exist. Systematically incorporating ethnicity, dietary culture, and individual variability into future study designs has therefore become an important direction for the field.

From the perspective of experimental models, most mechanistic studies currently rely on mouse models of DSS- or TNBS-induced colitis. Although these models can recapitulate acute intestinal inflammation, they cannot faithfully reproduce the chronic and relapsing disease course or the complex genetic background of humans with UC. Consequently, the actual expression levels, regulatory mechanisms, and therapeutic responses of molecular targets validated in animal models (such as the GPR37/ERK–NRF2–GPX4 pathway) in human intestinal tissues still require further confirmation.

At the level of research strategy, existing work has largely focused on single nutrients or bioactive components, such as arachidonic acid, n-3 PUFAs, and glutamine. However, in a real dietary context, complex synergistic or antagonistic interactions exist among various nutritional components, making the simple attribution of the observed effects to a single independent component difficult. Studies using whole dietary patterns, such as the Mediterranean diet and high-fat diet, as the entry point are still in their infancy. How the optimal ratios and dynamic regulation of various nutrients affect the prevention and treatment of UC remains to be systematically investigated.

Taken together, the limitations across these different levels point to a common underlying challenge: our current understanding of metabolic reprogramming in patients with UC still lacks critical evidence to bridge the transition from “molecular characterization” to “causal mechanism elucidation”. The molecular mechanisms identified in animal models are constrained by species differences and are difficult to translate into clinical practice; population-based studies, despite the use of prospective designs that approximate causality, still provide risk associations rather than causal validation; and multiomics technologies, while efficient at capturing UC-associated molecular features, mostly employ cross-sectional designs that offer only static disease snapshots, and thus distinguishing driver nodes from secondary changes within the metabolic reprogramming network is difficult. Therefore, future research must move beyond static, single-dimensional studies and integrate dynamic multiomics sampling of longitudinal cohorts with interventional studies to repeatedly track molecular changes within the same individuals across different disease stages and construct a complete causal evidence chain from “signal initiation” to “metabolic reprogramming” to “pathological progression.” These knowledge gaps highlight critical directions for future investigations and will also facilitate the development of precision dietary intervention strategies.

## 6 Conclusions and future perspectives

As a disease with complex phenotypes and an incompletely elucidated pathogenesis, the occurrence and development of UC are closely associated with metabolic disorders. Both epidemiological and basic research have indicated that dietary-derived substances and their metabolites play key roles in the UC process, not only exhibiting a close association with metabolic syndrome but also contributing to disease progression through multiple pathways, such as regulating immune responses, affecting intestinal barrier function, and remodeling the intestinal microenvironment. As one of the core features of UC, metabolic reprogramming influences the occurrence, development, and outcome of the disease.

Although the role of metabolic mechanisms in UC is increasingly recognized, this field still faces three major challenges. First, the metabolic regulatory network in UC is highly complex and involves the coordinated reprogramming of multiple cell types within the inflammatory microenvironment; conventional omics technologies are limited in their ability to comprehensively dissect this complexity at cellular resolution and in a spatial context. Second, extensive crosstalk exists between metabolic processes and other physiological modules, such as immunity and tissue repair, making causal relationships difficult to disentangle. Third, the metabolic differences between animal models and humans with UC hinder the clinical translation of basic research findings, and the metabolomic signatures of patients with UC have yet to be systematically characterized. Cutting-edge technologies such as single-cell transcriptomics and spatial transcriptomics represent critical breakthroughs to address these challenges. The former enables the dissection of metabolic reprogramming features across distinct cell populations at single-cell resolution, while the latter preserves the spatial context of gene expression, helping to reveal *in situ* interactions between metabolically dysregulated cells and infiltrating immune cells. Integrating these two technologies with metabolomics and metagenomics to construct a three-dimensional multiomics analytical framework will allow us to systematically map the metabolic remodeling landscape of UC from multiple perspectives—cell type, spatial location, and metabolic output—and to identify the key nodes driving metabolic reprogramming, thus providing a more precise theoretical basis for targeted therapy.

In terms of translational applications, modulating abnormal metabolic pathways has become an important direction for UC intervention. Based on the molecular characteristics identified using multiomics technologies, researchers can develop multitarget strategies to regulate metabolism and explore synergistic treatment modalities that combine metabolic interventions with immunosuppressants, consequently increasing efficacy and reducing the risk of drug resistance. More importantly, the management of the health of patients with UC may advance toward personalized therapy. By integrating multidimensional information such as the patient’s metabolic profile, microbiota structure, and immune status, a precision treatment system based on metabolic typing could be constructed. This approach may allow the customization of comprehensive management strategies for patients, including targeted metabolic modulation, personalized dietary plans, and scientifically informed nutritional patterns (such as intermittent fasting).
