# Correction: Metabolic reprogramming in ulcerative colitis: integrating dietary drivers, pathogenesis, and therapeutic advances

**DOI:** 10.3389/fimmu.2026.2010297

**Published:** 2026-10-14

**Authors:** Yunze Liu, Yisha Wan, Xinyu Lu, Aili Xu, Xia Ding, Yuan Li

**Affiliations:** 1 National Institute of TCM Constitution and Preventive Medicine, Beijing University of Chinese Medicine, Beijing, China; 2 The First Clinical Medical College, Xuzhou Medical University, Xuzhou, China; 3 Dongzhimen Hospital, Beijing University of Chinese Medicine, Beijing, China; 4 Wangjing Hospital, China Academy of Chinese Medical Sciences, Beijing, China; 5 School of Traditional Chinese Medicine, Beijing University of Chinese Medicine, Beijing, China

**Keywords:** diet, epidemiology, mechanisms, metabolic reprogramming, ulcerative colitis

Affiliation [1. National Institute of TCM Constitution and Preventive Medicine, Beijing University of Chinese Medicine, Beijing 100105, China] was erroneously omitted for author “Yunze Liu”.

The original version of this article has been updated.

